# Reflecting on Living Labs as Multi-Stakeholder Collaborative Networks to Evaluate Technological Products for People Living with Dementia

**DOI:** 10.3390/ijerph20031673

**Published:** 2023-01-17

**Authors:** Francesca Toso, Rens Brankaert, Niels Hendriks, Lieke Lenaerts, Andrea Wilkinson

**Affiliations:** 1 Human Centred Design (HCD) Group, Department of Design, Production and Management, Faculty of Engineering Technology (ET), University of Twente, Horst Complex, 7522 LV Enschede, The Netherlands; 2 Systemic Change Group, Department of Industrial Design, Eindhoven University of Technology, Atlas Building, 5612 AE Eindhoven, The Netherlands; 3 Health Innovations & Technology, Fontys School of Allied Health Professions, Dominee Theodor Fliednerstraat 2, 5631 BN Eindhoven, The Netherlands; 4 Interactions Research Group, LUCA School of Arts, C-Mine 5, 3600 Genk, Belgium

**Keywords:** design, living labs, technologies, dementia, home environment, field research, multi-stakeholder collaboration, scalability, certification

## Abstract

Dementia is a growing societal challenge putting pressure on care systems across Europe. Providing supporting technology for people living with dementia, referring to both people with dementia and their caregivers, is an important strategy to alleviate pressure. In this paper, we present lessons learned from the Interreg NWE Project Certification-D, in which we evaluated technological products with people living with dementia, using a Living Lab approach. Living Labs were set up in five different countries to conduct field evaluations at the homes of people living with dementia. Via an open call products from small to medium enterprises across northwestern Europe were selected to be evaluated in the Living Labs. In this paper, we describe the setup of and reflection on Living Labs as multi-stakeholder collaboration networks to evaluate technological products in the context of dementia. We reflect on the experiences and insights from the Living Lab researchers to execute and operate the Living Labs in such a sensitive setting. Our findings show that Living Labs can be used to conduct field evaluations of products, that flexibility is required to adopt a Living Lab in various care settings with different stakeholder compositions and expertise, and that Living Lab researchers serve as both a linking pin and buffer between people living with dementia and companies and thereby support the adoption of technological products. We close the paper with a proposal of best practices to encourage inclusivity in, and scalability of, Living Labs in the context of dementia.

## 1. Introduction

Over recent years, the number of people living with dementia, referring both to people with dementia and their caregivers, has increased continuously [1]. This puts pressure on care systems across Europe, and leads to challenges in offering humane care for people with dementia [2]. In addition, it is becoming more difficult to find suitable care personnel to provide care for the increasing number of people with dementia [3]. As a result, there has been a shift from providing care in specialized environments towards supporting people with dementia to live well at home. Therefore, it is necessary to find alternative and innovative ways to allow people living with dementia to live at home with a decent quality of life.

One of the ways we can support people living with dementia is by the use of technological products and accompanying services. These technological products can support people to live independently at home and offer support with communication [4], memory aids [5], outdoor orientation and navigation [6], facilitate social connection [7], support leisure activities [8], and more. However, supporting people with dementia adequately with technological products is challenging.

Specifically, technology to support people in their daily life activities is often referred to as assistive technology (AT), and encompasses a wide spectrum of technological products [9]. Challenges in adopting AT often come from a lack of proper involvement of the target group in the design process of the technology, causing these technologies to not properly meet needs [10]. In a recent review, a mismatch became apparent between the discourse of researchers, regarding the involvement of older adults in design processes, and the practice, where this only occurred sporadically and in a limited way [11]. It seems researchers and related developers of technology understand the importance of involving participants, but find it challenging to put this into practice.

Recent developments in HCI and design research have promoted an inclusive approach towards designing technology for people living with dementia [12]. Studies have shown the benefits of co-designing products with people living with dementia [13]. Also, the involvement of companies at an early stage during product design and evaluation allows for a better alignment with their needs [14]. While the methods and approaches to design technology are available, many products on the market are still not suitable for people living with dementia [15]. A recent review showed that people with dementia are often not involved in the early or late stages of an innovation or design process, causing challenges with regard to the adoption of the technology [16].

Living Labs are a suitable way to bring together and involve the various stakeholders that are required, such as people living with dementia, care organizations, knowledge institutions and companies, in the design of technological products [17]. Although Living Labs are used in different ways and there is no widely accepted definition of Living Labs [18], it is broadly understood as a means to conduct user research by means of co-design with the target group in a real world setting where relevant stakeholders are actively involved in the innovation process [19]. While studies conducted in a Living Lab might not be suitable to gain controlled results, they have been shown to offer high ecological validity [14]. Typically, studies conducted in a Living Lab setting gain insights from both qualitative and quantitative data sources to understand how people use a certain (technological) intervention. For many years, Living Labs have had a firm standing in Europe, especially since the European Union endorsed Living Labs under the European Network of Living Lab (ENOLL) as an important innovation mechanism to stimulate international collaboration to address societal challenges [20]. This paper presents a Living Lab project that was set up to evaluate and improve the design of technological products and services on the market for people living with dementia [21]. When people with dementia are involved as experts of their own experience, their input contributes to improve the quality, interaction design and adoption of technology [22]. The Living Lab allowed for the involved developers, researchers and participants to take different roles depending on the innovation needs and stage of the products [23].

To address the current challenges around products for people with dementia on a larger scale, organizations from five different northwestern European countries collaborated in the project Certification-D, funded by the European Union under the Interreg NEW program. Care organizations, knowledge institutes, companies and other public organizations collaborated to support small and medium-sized enterprises (SMEs) with an evaluation of their technological products in context, and provided input to improve the design of their product(s). If they fulfilled the necessary requirements, the project could certify the technology as being suitable for people living with dementia.

To do this, Living Labs were set up in each of the participating countries to allow people with dementia and their caregivers to use and experience the technological products offered by the SMEs. This was conducted following the Living Lab protocol as proposed for product evaluation in the context of dementia [17]. Following this protocol, the Living Lab was set up in a real-life setting of people living with dementia, either at their homes or locations were they come often such as a daycare center. This Living Lab operated in a distributed way, centrally organized by a care organization or public organization that was in close contact with communities of people living with dementia. These organizations partnered strongly with research institutes who supported the Living Lab research setup. The SMEs offered their products to be evaluated in the Living Lab, and in return they received feedback based on the experiences of the participants.

In this paper, we share what we learned from running five Living Labs within the context of dementia. We analyzed the comments and reflections from the main Living Lab researchers, who were key to the execution and operation of the Living Labs. Based on this, we offer best-practices and identify challenges when running a distributed Living Lab in such a sensitive setting. In addition, we reflect upon the multi-stakeholder collaboration between public organizations, care organizations and companies. Over the course of three years, 23 products from 20 SMEs were evaluated by 200 people with dementia. We show how the differences in setting, stakeholder composition, culture and discipline become apparent from the everyday practice of the Living Labs. We, therefore, recommend striving for a balance between structure and freedom for future Living Lab settings. In addition, we found that reflexivity and understanding of the dementia context is necessary to operate a Living Lab for such a specific target group.

## 2. Materials and Methods

### 2.1. Living Lab Approach

**Figure 1:**
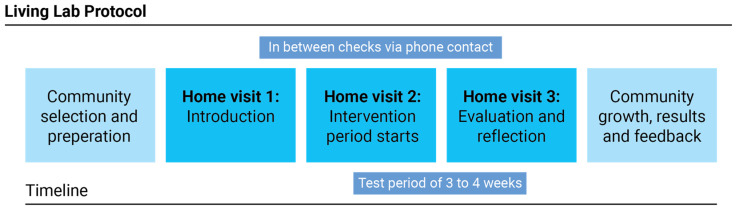
The Living Lab protocol used for evaluating interventions in context [17].

The five Living Labs in this project were set up as collaborative networks where the various stakeholders worked together to evaluate the technological products [24]. As such, the Living Lab structure/alliance allowed for individual Living Lab studies to evaluate products from SMEs. As a starting point, we adhered to the Living Lab protocol specifically proposed to evaluate interventions in the context of dementia [17]. The protocol allowed for user involvement and co-creation in the innovation process, which contributes to better products and higher adoption rates. This protocol had five stages: (1) community selection and preparation: in which the communities were informed regarding the project and participants were sought; (2) home visit, introduction: where the project and technology were introduced to the participants; (3) home visit, intervention: the start of the technology testing period; (4) home visit, reflection: when the intervention was retrieved, and the researcher and participant reflected and evaluated the experience; and (5) community growth, results and feedback: where the sharing of the results and outcomes contributed to the further growth of the community (Figure 1).

The Living Labs were set up as testing environments in which products from Small and Medium-sized enterprises (SMEs) were evaluated by people living with dementia. The evaluation took place in the homes of people living with dementia, either independently, with informal or professional caregiving support, or during a daycare program for people still living at home. The use period was six to eight weeks, and support from the Living Labs and companies was available if needed.

After the evaluation period, we collected qualitative and quantitative data regarding the user experience and appropriateness of the product for people living with dementia. The evaluation was performed with a questionnaire that was based on product requirements and qualities collected via a Delphi study [25] and on the experience of researchers with products for the care and support of older adults. The aim of the questionnaire was to gather data needed to set up a scoring system to provide a certification mark to SMEs when the results were sufficient. In addition, the questionnaire allowed for open answers contributing to understanding the user experience.

### 2.2. Living Lab Practical and Ethical Organisation

In practice, the Living Labs were organized as a collaboration between care organizations and research institutes with experience in working with people living with dementia. The care organizations ensured access to the target group and contact via care professionals, to include participants in separate Living Lab studies on a voluntary basis.

All the studies were conducted in compliance with the ethical regulations that applied for each of the collaborating institutes in each of the five Living Labs. The connected universities applied for ethical approval of the Living Lab setup and studies at their own organization. The guiding principle for this approval was the safety and the privacy of the participants and the voluntary consent of participants. All participants could stop at any moment, and no financial involvement was asked nor provided to them for this study. If the participants enjoyed the use of the product/service that they tested, they received the contact details of the company and, in some cases, a discount to purchase the product.

Following the proposed Living Lab protocol [14], different types of partners were involved in each of the Living Labs. In total, five Living Labs were set up: in Belgium (University’s art and design faculty and a care organization), France (an expertise center with their own partners), Germany (a mental care hospital and an in-house research center), the Netherlands (a care organization in collaboration with a technical university) and Northern Ireland (a technical university with their partners).

### 2.3. Process of Involving SMEs and Selecting Their Products

In the project, we included SMEs in two phases, indicated as ‘waves’. In each wave, SMEs could apply for a voucher to have their product evaluated by people living with dementia in the Living Labs. For each wave, we held a public call for participation targeted at SMEs. The call was distributed online [25] via the project website, social media and in the newsletters of each project partner. In addition, events were organized to explain the call and answer questions from SMEs.

The selection of the SMEs took place under the supervision of a committee consisting of different partners (lead partner, partner responsible for organizing the waves, and additional members), and was based upon predefined project requirements, such as: (1) dimensions of the company (SMEs), (2) location (in northwest Europe), (3) technology readiness level above level 6 (as defined by the ISO 16290:2013 [26], adopted as a reference scale from the European Union for the Horizon 2020 program in 2014), and (4) product category (technical products or services for people with dementia to be used at home). To further streamline the selection, we applied the following exclusion criteria: (1) no medical devices or edible products (these were outside the scope of the project and would have required expertise outside of the consortium, as well as additional implementation regarding ethical considerations), and (2) no major financial support received from public entities in the previous year (EU regulations). Companies deemed eligible based on the selection criteria were invited to apply to the project. Based on the outcomes, a selection of products was proposed to be evaluated in the Living Labs.

After selection, the partner responsible for the process of involvement of the SMEs proposed how to divide the products across the five Living Labs. The proposal, based upon the qualities of the products and the expertise of each Living Lab, was discussed with the Living Labs and adapted, taking into account the preferences and options of each Living Lab. At this point, the SMEs that were selected were put in contact with the Living Lab(s) in which their products would be tested: the SME and the Living Lab(s) signed a cooperation agreement, which attached a document defining the testing set-up.

The call for wave 1 was launched in November 2020 and the involvement of SMEs terminated in December 2021. We received 19 applications via the sign-up form, of which 18 SMEs were eligible and invited for interview. As a result of the selection, we invited 10 SMEs with 12 products to test in the Living Labs. One SME dropped out following acceptance into the Living Lab program, since their product did not work as promised.

The call for wave 2 was launched in June 2021 and the involvement of SMEs terminated in December 2022. We received 23 applications via the sign-up form, of which 18 SMEs were eligible and invited for interview. As a result of the selection, we invited 12 SMEs with 13 products to test in the Living Labs. One SME dropped out before testing occurred since their product could not be installed at one of the Living Labs.

Eventually, 9 SMEs with 11 products participated in voucher wave 1, and 11 SMEs with 12 products participated in voucher wave 2, being a total of 20 companies involved with 23 products.

To close the collaboration between each Living Lab and the SMEs in the project, all companies involved received a formal closing email from the Living Lab in which their product(s) were tested. The email included a letter declaring the conclusion, reporting the details of the testing, and an invitation for an online outro interview to reflect on their experiences.

### 2.4. Certification Procedure and Closing the Living Lab Participation

After a pilot round to set up the Living Labs, wave 1 was conducted to verify the testing process, the data collection and the selection procedure. Wave 2 was set up as a definitive process, and granted access to the certification procedure and the certification mark for the SMEs that met the requirements.

The questionnaire we previously introduced played a relevant role in the collection of the results from the Living Labs: the questionnaire covered quantitative responses necessary to establish an objective product evaluation, and qualitative responses used as feedback for the companies and as grounds to improve their product further.

Questionnaires were filled from the perspective of the person with dementia, often supported by an informal or formal caregiver. Each questionnaire was structured in three parts: (1) general information, (2) questions about the product, and (3) further questions, and was meant to be used as a paper questionnaire printed on A4 sheets. For most quantitative questions, the participant could reply on a scale from 0 to 4, by means of emoticons ranging from smiling to sad. The qualitative open answers were collected in the form of descriptive text at the end of each section.

A minimum number of questionnaires (10) from people with dementia about a specific product were required by the certifying body to access the certification process. The questionnaires addressed the usage of the products in terms of: (a) product interaction, (b) product quality, and (c) accessibility from the perspective of the person with dementia. A grade was formulated based on the quantitative result from the questionnaire. The calculation conducted by the certifying body defined the threshold for the product to obtain the certification mark Certification-D. This section has described the procedure of the certification, since we will reflect on the experience of conducting these evaluations by the Living Lab researchers. The data from the questionnaires themselves is not used or presented further in this study.

## 3. Data Gathering for This Study

For this paper we focused on collecting *written reflections* from the researchers involved in the Living Labs. We invited the main coordinators to provide us with feedback and reflections on their experience in the Living Labs, allowing us to compare and distil overarching insights. We collected reflections from one or two researchers—the main researchers—in each of the Living Labs. In total, this accumulated to nine researchers. These Living Lab researchers supported in: (a) executing the protocol (see Section 2), (b) arranging participants and company contact, (c) delivering and assisting with products where needed, (d) retrieving products and taking questionnaires at the end of testing, and (e) de-briefing with the SMEs to support the innovation process.

After each product evaluation in the Living Labs, the researchers from each Living Lab filled in a reflection form to report on their experience during the test. We received input from all five Living Labs. The form addressed the evaluation process (based on the separate steps in the Living Lab protocol), the collaboration with the SMEs, and the involvement of participants and colleagues. In addition to providing feedback to the project to improve the procedures, these reflections were also shared with the SMEs.

The data collected with the Lessons Learned documents were translated into short quotes and statements that were collected using Miro software and then clustered by the first author. The goal of the cluster exercise was to find differences, commonalities and surprising topics from the collected data. In the first step, a mapping exercise was conducted, the statements were written as individual digital notes on the Miro board, and tagged with the call wave (1 or 2) and the particular Living Lab researcher making the statement. After a first screening of the data, the notes were redistributed and divided into themes. The themes identified, and the outcomes of the analysis, are presented in Section 4.

## 4. Results

In this result section, the topics and themes resulting from the cluster exercise are presented. Based on the qualitative reflection data from the Living Lab researchers we formed three topics:

1. Topic 1: involvement of participants and SMEs in the Living Labs;
2. Topic 2: operating the Living Labs following the protocol;
3. Topic 3: participating in the Living Labs.

### 4.1. Topic 1: Involvement of Participants and SMEs in the Living Labs

While the Living Labs were based on the same Living Lab protocol, the practical implementation of each of the Living Labs was different, which influenced the recruitment strategy and the testing situation.

Each Living Lab initiated recruitment following their own capacity and stakeholder setup. All projects recruited participants via care organizations, however, for two Living Labs, the care organizations were project partners (internal), and for the other Living Labs, the care organizations were outside the project (external). Working with external organizations was found to be more difficult to collaborate, since the researchers had to first build a trust relationship. In all cases, professional caregivers supported the finding of suitable participants based on their own judgement and the requirements of the products.

In general, it was difficult to recruit participants for evaluation: some products required the participants to have specific skills, some had specific installation requirements that did not fit with the participants’ living environment, some required the presence of (in)formal caregivers and their willingness to invest time in the product testing, as well: “*In the beginning it was difficult to recruit participants, but at the end it was easy. I think it just takes time to make people enthusiastic*” *(from Lessons Learned report)*. In addition, the recruitment of participants was perceived as a time-consuming process: “*The problem is that district nurses have to make the first contact with the potential participants. They do not have or do not take the time for this.*” *(From Lessons Learned report)*. Finally, the COVID-19 pandemic played a huge role during the project, and made recruiting participants even more difficult.

Furthermore, the language in which the products and their supporting materials were provided introduced a further communication barrier: not all the participants were able to understand or speak English. A recurring comment from the care professionals was the need for receiving products in the local language: “*Also the [name of the product] was all in English and translation took a while. That was a shame and it made recruiting hard.*” *(From Lessons Learned report)*. However, sometimes the companies stepped in and supported the required language: “*There were only manuals or flyers in English. [Name of the company] made a short instruction manual in Dutch for the participants.*” *(From Lessons Learned report)*.

The Living Lab researchers dealt differently with the products they received from SMEs. Some Living Lab researchers brought the packages as received from the companies to the participants, and left the unpacking to the participants. While this provided an authentic experience for the participants, it was often challenging to understand the products. In one Living Lab, this was addressed by introducing the products in a communal setting, contributing to a shared understanding of the functionality. In other cases, companies provided training to the Living Lab researchers to instruct them on the appropriate use of the products and their functionalities, which contributed to a proper adoption of these products. “*The contact with the SME was really good in the beginning. They gave a presentation and explained everything, they really made time for it.*” *(From Lessons Learned report)*. During some evaluations it was difficult to contact the companies and to obtain support. In these cases, the delays in receiving assistance caused frustration and even drop-outs in participants. However, most SMEs were motivated to evaluate their product and supported the Living Labs thoroughly: they kept regular contact with the researchers, provided additional explanatory material if needed (sometimes they translated it if needed), offered online and offline training, and gave assistance when technical issues occurred.

During testing, the Living Lab researchers took on a more intermediate role between the company and the participants. This worked well to translate intentions back and forth, however, it was also difficult when agreements were not followed through. For example, the online training for one provided product, was not enough: “*[Name of the product] is a product with lots of features, which I don’t know how it all works. This makes it hard to explain the right way to the participants. An online training is not enough for this.*” *(From Lessons Learned report)*. Additionally, people working as professional caregivers are under pressure and might not have a large amount of time to support product adoption in Living Labs, which could result as a tradeoff in the Living Lab: “*The instruction from [person] was clear. Afterwards my colleagues said they thought it was a bit too long. It could have been a shorter introduction, also because it’s not difficult to use. I thought it was nice to hear how the product is developed and the meeting created some extra motivation to use and involvement of [person]. It made it easy to contact him.*” *(From Lessons Learned report)*.

### 4.2. Topic 2: Operating the Living Labs following the Protocol

The Living Lab protocol was sufficiently open to adapt to the individual needs of each Living Lab and the abilities of the Living Lab researchers. For example, one Living Lab researcher adapted the format of the questionnaire to question cards enhanced with images to improve the understandability of the questionnaire. In another Living Lab, the first part of the protocol was adapted as some participants felt the first meeting was overwhelming. Their new proposal was more time consuming but worked well for both the Living Lab researcher and the participants: “*The first participant was overwhelmed at the first contact. Later we adjusted the first contact moments. We did not immediately go in with the product, but first made informal contact. Even with coffee and cake. We only started talking about the project later. This went better.*” *(From Lessons Learned report)*.

While operating the Living Labs, the researchers encountered different cases in which the technical requirements of a product were not met by the living environment of the participants, for example, due to a lack of internet connection, devices not being compatible with the electrical system of a specific country, participants did not own the required support devices such as tablets or smartphones, or the application did not run on the participants’ existing devices. Different solutions were adopted to overcome these challenges in the Living Lab. In these cases, SIM cards, internet connection, smartphones and tablets were provided for the duration of the testing by the SME, or products were substituted with alternative solutions.

The Living Lab protocol suggested in-between checks to stay in touch with the participants and support if needed, and each Living Lab decided how to do this on their own. In one Living Lab, the researcher kept regular text message contact with the family members of the people with dementia; in another case, the researcher provided their own contact to be used if needed. Another researcher checked in with family members and care staff, but not regularly: “*For the periodical checks we went in contact with the group staff to see if everything was clear.*” *(From Lessons Learned report)*. All researchers were available in case of issues with the products, if participants considered dropping out, or when any kind of assistance was needed: “*In the [Living Lab], we don’t organize periodical checks, however people can always get in contact with the LL reference person (if needed)*.” *(From Lessons Learned report)*. Participants reached out to the researchers when, for example, they missed or received too frequent alarm signals, or when technical difficulties occurred during the setup of the product; in other cases, the researchers did not hear from the participants until the last meeting.

Following the protocol, a final meeting was set up to collect the product, in the cases of testing at home, and to fill in the questionnaires to evaluate the products. Some participants were autonomous in filling in the questionnaire without the supervision of the researcher—the (in)formal caregivers filled it in based on the answers of the people with dementia, and gave it back to the researchers; the caregivers were instructed to let the person with dementia fill in the questionnaire by themselves and help them only if it was too difficult or frustrating for them.

In other cases, the researchers used the template to conduct a reflective, in-person interview with the person with dementia and the (in)formal caregiver, and filled in the questionnaire at their home. In the case of the Living Lab that distributed the questions in the form of cards, the filled-in cards were collected. When the cards were not filled, the researcher filled them at the time of collection by starting a conversation with the person with dementia and the caregiver(s). When the testing took place in a care home setting, such as a daycare center, the questionnaires were filled in during a group session. The questions were brought in for the whole group to build a conversation around the testing experiences: “*(…) the questionnaire was filled out in the group room with every participant. The participants liked the idea, but filling out the questionnaire seemed hard for them.*” *(From Lessons Learned report)*. The Living Lab researcher, however, noticed that the participants ended up influencing each other’s answers and opinions and decided to switch to one-to-one interviews: “*We tried some different ways to fill out the questionnaire. Best way was to speak to each person alone and read out the questions. If something was not clear, we could ask the question again or they could ask for an explanation. It was the most time consuming method but it was the most effective. One other way we have tried was to give the questionnaire to each person and they filled it out by themselves. This was also okay and worked fine. One time we gave the questionnaire to more than one person at the same time. Everyone filled out their own questionnaire, but we noticed they were chatting all the time and consulting so we intervened. Personally, I think it is a good way to get more information, but it most likely would bias the individual answers of the questionnaire.*” *(From Lessons Learned report)*.

Some Living Labs adopted the practice of bringing a gift to the participants at the end of the testing to thank them for their participation. “*After filling in the questionnaire together with a researcher (…), [product name] was collected and each test person or daycare centre received a small token of gratitude (for example leftover products from VW1 and VW2)*.” *(From Lessons Learned report)*. Other gifts varied from products designed specifically for people with dementia (coloring books, set of cards), to discounts for the products participating in the voucher waves: “*Discount codes of all products were mailed to each participant in the first voucher wave*” *(from Lessons Learned report)*.

### 4.3. Topic 3: Participating in the Living Labs

Participants in the Living Labs were involved by the Living Lab researchers, often in collaboration with a care organization. While the project partners were involved in the selection of products to test in the Living Lab, it would be preferable to involve the carers and people with dementia directly. “*A more secure selection of the products would be nice. For example involving carers, family members or the people with dementia themselves to experience the product. Then they test the product because of their own motivation and believe in the product and not because we ask them to.*” *(From Lessons Learned report)*.

The criteria for involvement targeted people living with dementia living at home, who are difficult to reach in general. Therefore, care organizations supported the Living Lab researchers by directing them to people with dementia that would benefit from certain products, or people they expected to be enthusiastic and open to participating in the testing. The project focused on people with dementia for their testing, but the informal and formal caregivers had a strong influence on participation. This happened both ways. Sometimes, their motivation and willingness to try a specific product helped to convince the person with dementia to participate: “*All this effort resulted in only one more test person, however she was the grandmother of the LL intern so instead of wanting to test the app, she tested the app to do her granddaughter a favor*” *(from Lessons Learned report)*. In other cases, the attitude of the family members caused the people with dementia to hesitate to participate in the project. “*The caregivers understood the project [name], but I noticed they were more interested in testing the product and the added value of the product for the participant or themselves.*” *(Lessons Learned report)*. In most cases, the informal caregivers were the main point of contact for the researchers as they facilitated the Living Lab participation and use of the product. They were often eager to provide their feedback at the end of the testing, which could sometimes influence the results towards their perspective: “*The questionnaires were all administered to the participants themselves in the presence of the carers. It is difficult to ensure that you only get answers from the participant. The carers are quickly inclined to have their say*” *(From Lessons Learned report)*.

The quality and type of products affected the participation too. Some products that were deemed too complex were not easily accepted; similarly for products that required continuous effort from relatives. In addition, some product failures, such as false alarms, caused anxiety in the participants, resulting in diminished interest and reduced use of the devices, minor damages to the products, and in some cases, drop-outs: “*They really liked the idea of the product, but they had also a few false alarms, which made them feel insecure about the product*” *(from Lessons Learned report)*. Additionally, products perceived as stigmatizing were sometimes not accepted by participants; and when recognized as useful, were used with limitations: “*One tester liked the idea of the product but would have preferred if it was less noticeable when worn—didn’t like wearing in public. One tester didn’t wear it in the bathroom or when getting ready.*” *(From Lessons Learned report)*.

## 5. Discussion

In the project, the Living Lab researchers evaluated many different products from SMEs in the homes of people living with dementia. The Living Labs were centrally set up starting from the same Living Lab protocol, but resulted in five unique Living Lab settings over the course of the project following local needs, abilities and stakeholder composition. By collecting the reflections from the Living Lab researchers who ran these Living Lab evaluations, we found best practices and challenges in the operation of Living Labs.

In the following sections, we will discuss our experiences regarding Living Labs to support future technological product evaluation in the context of dementia.

### 5.1. The Product Categories and Qualities from SMEs

In the project, we conducted an open call for the inclusion of products from SMEs from different countries from the northwestern European region to be evaluated in the Living Labs. Therefore, the resulting series of included technological products varied widely. This made it difficult to manage expectations by Living Lab researchers towards participants, and they had to continuously adept. Additionally, the array of products each had different requirements, which made them sometimes challenging to cater for in the Living Labs. Other issues that came forward were due to company challenges such as set deadlines of delivering products not being met, or technical preparation not arranged properly. For example, SMEs only offering their product on one operating system, or basic technical support not being available, such as a internet connection, specific smart devices, or versions of smart devices. In the case of testing digital applications, for example, where third party hardware was required such as a smartphone, it was hard to distinguish in the evaluation between the hardware and the software. This illustrates and re-enforces that SMEs need a basic understanding of the context where their products will be deployed, in this case, dementia. We found this was often lacking; some SMEs were so focused on their own product and making their own product function, that they could not see the evident shortcomings to make it work for people with dementia. Providing a basic level of training, and insights into the first-person experience of dementia, would contribute to a better adoption of the products [27]. Different experiences of co-creation have reported the need for developing individualized solutions to empower people living with dementia [28], addressing both physical needs and psycho-social well-being [29], and involve multiple stakeholders over the course of the entire product design and development process to overcome adoption challenges [30].

While we had inclusion and exclusion criteria, it was still not always what SMEs meant. For example, some SMEs offered only partly functional products, or products that did not comply with the required product maturity of technology readiness level 6 or higher (see Section 2.3). The Living Lab researchers had to follow up with each company specifically to discuss terms and options. Based on our experience, we would propose a stricter selection procedure in the future, where unsuitable products can be filtered out more easily, and to place the burden on the researchers while operating the Living Labs.

The five Living Labs were located in Belgium, France, Germany, the Netherlands and the United Kingdom, based on the type of product and preferences from both SME and Living Lab researchers. For some SMEs that only operated locally, allowing their product to be tested and used by people living with dementia in another country was interesting but also caused challenges. The most obvious challenge was language, as people living with dementia often only speak and understand their own native language, but also other issues occurred such as power net requirements and the incompatibility of international communication networks (e.g., SMS with info on alarms were not accepted by international phone numbers). We found that there was sometimes a mismatch between SMEs’ ambitions and what they could deliver, that should be monitored carefully in the future.

### 5.2. Collecting and Communicating the Results

The researchers involved in the activities of the Living Labs were critical in conducting the questionnaires to evaluate the experiences with the product. Aspects such as the language used, the length, and the format, were perceived as not appropriate for the target group and they were adjusted where possible. It is recommended that questionnaires are co-designed with people with dementia, taking into account experiences from researchers in other fields on gathering qualitative data in sensitive settings [31,32,33]. In practice, the Living Lab researchers adapted the method of administering the questionnaire to fit the dementia context. This, however, challenged the validity of the questionnaire to produce an objective outcome. For example, differences arose because Living Lab researchers had different backgrounds, levels of expertise in conducting research, and different hosting structures for administering the questionnaires (for example, group vs individual). In addition, changes in personnel within the Living Labs also caused differences in how the evaluations were conducted between products.

To offset these differences, regular meetings were organized by the Living Lab researchers to calibrate their approach. However, these meetings often became a reporting occasion, focusing on the exchange of general information and light updates. Here the need to be flexible to act within the context of dementia was challenged by the time pressure experienced within the project, a common experience in research practice in this domain [34]. To overcome this and maintain a healthy research environment, we advise that Living Lab researchers be brought together regularly to discuss the execution in more detail, in order to build a group experience, address and solve problems that occur within individual Labs, and offer shared training on how to conduct the Living Lab evaluations.

The involvement of participants in the Living Lab was closed on completion of the questionnaires and collection of the products. However, a good practice would be to set up an exit strategy in conducting research with people living with dementia [34,35]: the participants involved in the testing were curious about the results, and therefore it is important to report back to them what happened with their input.

### 5.3. Involving People Living with Dementia in the Living Labs

The Living Lab researchers reported that participants felt a sense of purpose and curiosity to try out new products, contributing to their willingness to join. The Living Lab researchers actively looked for suitable participants for each product, which could have a caused a positivity bias, meaning that the apparent match could have resulted in a more positive evaluation of the products. However, the results showed varied outcomes, from very positive to very negative. This confirmed that a random selection of participants would not make sense with such a varied array of products, as some products would clearly not work for certain participants [36].

The Living Labs, being organized at a local level, allowed for more flexibility in their structure, sustaining a person-centered approach, thus, allowing the adaptation of the protocol to the participants and SMEs when needed. This made the overall inclusion of participants less representative, since the participants were always recruited via the same care organizations connected to the Living Labs. In addition, since no specific personal data was gathered, we could not draw additional conclusions or suggest how additional factors such as cultural background, financial situation, family status, age or gender would influence the adoption of the technological products by people living with dementia more general. For example, different studies have addressed the impact of gender and cultural background in the adoption of assistive technologies [37,38]. We recommend investigating these factors further in future research.

In undertaking research with people with dementia, we applied a person-centered approach that aimed to address the personhood of the participants instead of identifying them with their illness. This perspective provides more subjective outcomes, focused on the experiences of a reduced number of participants with dementia. This is important since the course of dementia can vary for each person in an unpredictable way. To address this, emphasis was placed on involving people with dementia in a person-centered way, considering the change of the condition over time [39]. This however meant that the results were not directly representative for the entire population, but did provide a good sense of how the product performed in context.

As shown in other cases [40], project-related parameters can pose additional challenges to conduct research in the context of dementia, and cause a too-narrow focus on, for example, early stages of dementia. For further studies, the initial requirements can be expanded to provide a more inclusive representation of people with dementia in different stages.

### 5.4. Scalability of the Living Labs

The five Living Labs operated in two call waves, evaluating 23 products in total. By distributing the products between the Living Labs based on interest and ability, a balanced evaluation was conducted. There were, however, some challenges in conducting the Living Lab methodology appropriately and finding sufficient participants for each Living Lab evaluation. These aspects make it difficult to scale up the Living Labs, and need to be addressed in further research.

The Living Lab researchers found it challenging to recruit the required ten participants per product (even when distributed), hampering further scaling and adoption of the Living Lab approach by other projects. Some activities in the Living Lab were time intensive, and will need to be re-adjusted when scaling up to a larger number of products. For example, the capacity of a daycare center is limited, care organizations only have access to a limited number of households, and Living Lab researchers can only maintain contact with a limited number of people and companies.

The differences in the context of the Living Labs required flexibility in the application of the protocol. Following the protocol, the Living Lab researchers needed to find a balance between being open enough to allow custom adaptation of the Living Lab in line with regional requirements, and being sufficiently structured to allow comparison of results. We suggest an implementation of the system to strengthen the Living Labs based upon the evaluation structure proposed in the VITALISE project [41], and to provide specific training for the organizers of Living Labs in the context of dementia. In particular, the adoption of training programs shared with a bigger network composed of more mature Living Labs could contribute to strengthen the structure of the Living Labs, foster a reflective mindset and promote continuous self-assessment.

Finally, over the course of the project, there was a continuous tension between a market orientation, supporting SMEs to make better products and, thus, generate more economic revenue, and a user orientation, involving people with dementia to provide a better experience. These need to be in balance. In a project such as this, it is important to explicitly state the values and priorities underlying the project decisions, since these might differ per involved organization. When scaling up, it is important to ensure that there is a sustainable plan underlying the Living Lab, allowing it to generate sufficient value while also conducting studies ethical, careful and sensitive to the context.

## 6. Conclusions

The project Certification-D brought us insight into how to conduct a practical Living Lab with real products from SMEs in a real-life setting of people living with dementia at home. Operating a Living Lab in such a dynamic context contributes to the ecological validity of the research, however it causes various challenges such as dealing with the variety of products, representativeness of results, preparation of the participants, stakeholder alignment and scaling up of activities. We formulated the following conclusions from reflecting on the experiences of operating the Certification-D Living Labs.

Conducting an activity with the Living Lab methodology requires time and commitment from the institutions involved. Sufficient time for recruitment, therefore, needs to be provided by these organizations. The many various activities that are needed to conduct a Living Lab study need to be foreseen by all partners and especially companies, such as the delivery of products to and from the Living Lab, eventual training or installment, and support when issues occur.

In addition, flexibility is required by the Living Lab researcher involved. The implications of living with dementia need to be acknowledged and respected by care professionals, researchers and companies. This could mean the evaluation requires multiple meetings to explain and repeat the instructions, that evaluation time sometimes needs to be extended, or that methodologies must be adapted to improve accessibility.

The benefits for the care organizations to participate in the Living Labs, was mainly to have first-hand experience with new innovative technologies before committing to buy them. As such, the field-testing of products becomes a co-design environment for companies, in which products can be customized to the needs of the people and organizations involved. In order to facilitate this, the Living Lab researchers should receive proper training on both conducting such studies and on how the products work. If so, they would be able to better match people living with dementia with the products, allowing them to become a stronger intermediary between the different stakeholders involved.

The process of evaluating products in a real-life setting is time consuming and also requires full commitment from the SMEs. For an optimal process, the SMEs should provide assistance, ensure that products and materials are delivered in good order, provide training, and offer their service in the local language. These concepts are especially important when operating in a sensitive setting. The main benefit for the SMEs was the access to, and direct contact with, people living with dementia. The Living Lab supported them in testing their products, and as such, contributed to their product development, as well as providing them with a supportive group of experienced researchers. Overall these insights contribute to design more suitable products for people living with dementia, and as such contribute to the challenges of dementia for our society.

## Data Availability

Not applicable.
